# Sex differences in speed and sub-technique selection in elite sprint cross-country skiers: a time-trial qualification analysis

**DOI:** 10.3389/fspor.2026.1845432

**Published:** 2026-06-17

**Authors:** Øyvind Sandbakk, Jan Kocbach, Rune Kjøsen Talsnes, Thomas Losnegard

**Affiliations:** 1 School of Sport Science, UiT The Arctic University of Norway, Tromsø, Norway; 2 Department of Physical Performance, Norwegian School of Sport Sciences, Oslo, Norway; 3 Centre for Elite Sports Research, Department of Neuromedicine and Movement Science, Norwegian University of Science and Technology, Trondheim, Norway

**Keywords:** classical technique, cross-country skier, cycle length, sprint skiing, XC skiing

## Abstract

**Purpose:**

This study investigated sex differences in speed, sub-technique selection and cycle characteristics during a sprint cross-country skiing time-trial qualification in the classical style.

**Methods:**

Thirty elite- to world-class cross-country skiers (15 women, 15 men; mean International Ski Federation [FIS] sprint points ∼58 in both groups) performed an FIS-regulated on-snow classical sprint competition. The initial 1.3-km time-trial qualification (prologue) was analyzed using a combined global navigation satellite system and inertial measurement unit to determine speed, sub-technique selection, and cycle characteristics.

**Results:**

The men were ∼14% faster than the women (mean speed: 8.26 vs. 7.23 m·s^−1^, *P* < 0.001). The sex difference in speed was greatest in the uphill sections (19%–20% difference) and smallest in the downhill sections (6%–9%), with comparable differences in flat terrain (∼13%–14%). The men spent larger portion of the time trial using double poling than the women (∼61% vs. ∼53% of total time, *P* < 0.001), whereas women used diagonal stride more than men (∼23% vs. ∼16% of total time, *P* < 0.001). Cycle analysis revealed that the men had 9%–11% longer cycle lengths than the women in both diagonal stride and double poling (both *P* < 0.05), while cycle rates were generally similar between sexes.

**Conclusion:**

Elite male cross-country skiers were ∼14% faster than performance-matched female skiers during a sprint time-trial qualification with equal distance. The most notable difference appeared on uphill terrain, where men relied more heavily on double poling and achieved longer cycle lengths across all sub-techniques. Consequently, female and male skiers face distinct sport-specific demands when competing over equal distances.

## 1 Introduction

Sprint cross-country skiing is a physiologically and technically demanding endurance event involving repeated high-intensity efforts over short distances (∼1.5 km), starting with an initial time-trial qualification followed by three subsequent knock-out heats (1, 2). These repeated efforts involve continuous transitions between different terrain segments, making optimal pacing and sub-technique selection crucial for performance (2–5). In addition to a high aerobic energy turnover and efficient skiing technique, the constant changes in terrain segments and exercise intensity place unique demands on the interaction between substantial oxygen deficits in uphill segments and rapid recovery in subsequent downhill segments (2, 3).

While sex differences in many endurance sports are well-documented (6, 7), limited research has explored such differences in sprint cross-country skiing, and particularly when performance-matched male and female skiers compete over identical distances. Previous studies indicate that male cross-country skiers exhibit 10%–16% higher average speeds than female skiers in distance competitions above 5 km with the most pronounced differences seen in uphill terrain segments (8–11). These differences are explained by disparities in maximal aerobic power ($\dot{V}$O_2max_) muscle mass, and strength, particularly in the upper body, between men and women (6, 12–14). Accordingly, this influences both speed and technical solutions, as male skiers tend to utilize high-speed sub-techniques more frequently, whereas female skiers rely more on lower-speed sub-techniques (8, 10, 11, 15). Since most previous studies have focused on distance competitions (8–11) and sprint competitions in the skating technique (15), research exploring how these differences translate into the classical technique during sprint time-trial qualifications remains scarce. This update is particularly important for elucidating sex-specific demands, given that men and women now compete over identical distances.

Recent advancements in wearable sensor technology, such as high-precision global navigation satellite systems (GNSS) and inertial measurement units (IMUs), allow continuous monitoring of speed profiles, sub-technique selection, and cycle characteristics throughout entire racecourses (16). These tools provide unprecedented opportunities to compare real-time performance characteristics between male and female skiers across varying terrains, enabling a more detailed understanding of sex-based differences in sprint cross-country skiing. This could contribute to increased understanding of sex-specific demands and be helpful for coaches when optimizing individual training and competition strategies for both male and female skiers.

Therefore, this study aims to examine sex differences in speed, sub-technique selection, and cycle characteristics during a sprint cross-country skiing time-trial qualification over an identical distance using the classical technique. By utilizing state-of-the-art tracking technologies and performance-matched elite male and female skiers, we provide novel insights into the technical and physiological factors contributing to observed sex differences in sprint cross-country skiing.

## 2 Materials and methods

### 2.1 Participants

Thirty (15 women, 15 men; average International Ski Federation (FIS)-points before the competition: 58.9 for men and 57.7 for women; age 26 ± 4 years) elite- to world-class Norwegian cross-country skiers participated in the study. FIS-points and ranking data from the analyzed competition is included in Table 1. All participants were recruited from national and regional teams within the Norwegian Ski Federation. The study was approved by the ethics committee at the Norwegian School of Sport Sciences (ref: 135-180620 and 285-150623), deemed advisable by the Norwegian Center for Research Data, and conducted in accordance with the Declaration of Helsinki. All participants provided written informed consent prior to participation.

**Table 1:** Participant characteristics including their FIS points and the rank obtained during the present sprint time-trial competitions in elite- to world-class male and female cross-country skiers.

Variable	Statistics	Women (*n* = 15)	Men (*n* = 15)	*P*-value	ES
FIS Points	Mean ± SD	68.7 ± 24.5	68.5 ± 24.8	0.99	−0.01
	Range	30–105	22–101		
Rank	Mean ± SD	10.9 ± 5.8	25.3 ± 17.1	0.007	1.12
	Range	2–20	1–52		

### 2.2 Study design

The participants performed a FIS-regulated individual sprint competition in the classical technique at the beginning of the competition season (November 2022). The competition was performed in a homologated sprint racecourse (∼1.3 km) and only the initial time-trial qualification (prologue) was used for analysis. The participants’ speed, sub-technique selection, and cycle characteristics (cycle length; CL and cycle rate; CR) were continuously measured during the time-trial qualification. The racecourse included segments of uphill, flat, and downhill terrain, with precise measurements obtained from GNSS data. All participants used their own ski equipment, including poles, boots, and skis, prepared according to their individual preferences. In all analyses, male and female skiers were performance-matched by use of the FIS points obtained from the competition.

### 2.3 Weather and course conditions

**Figure 1:**
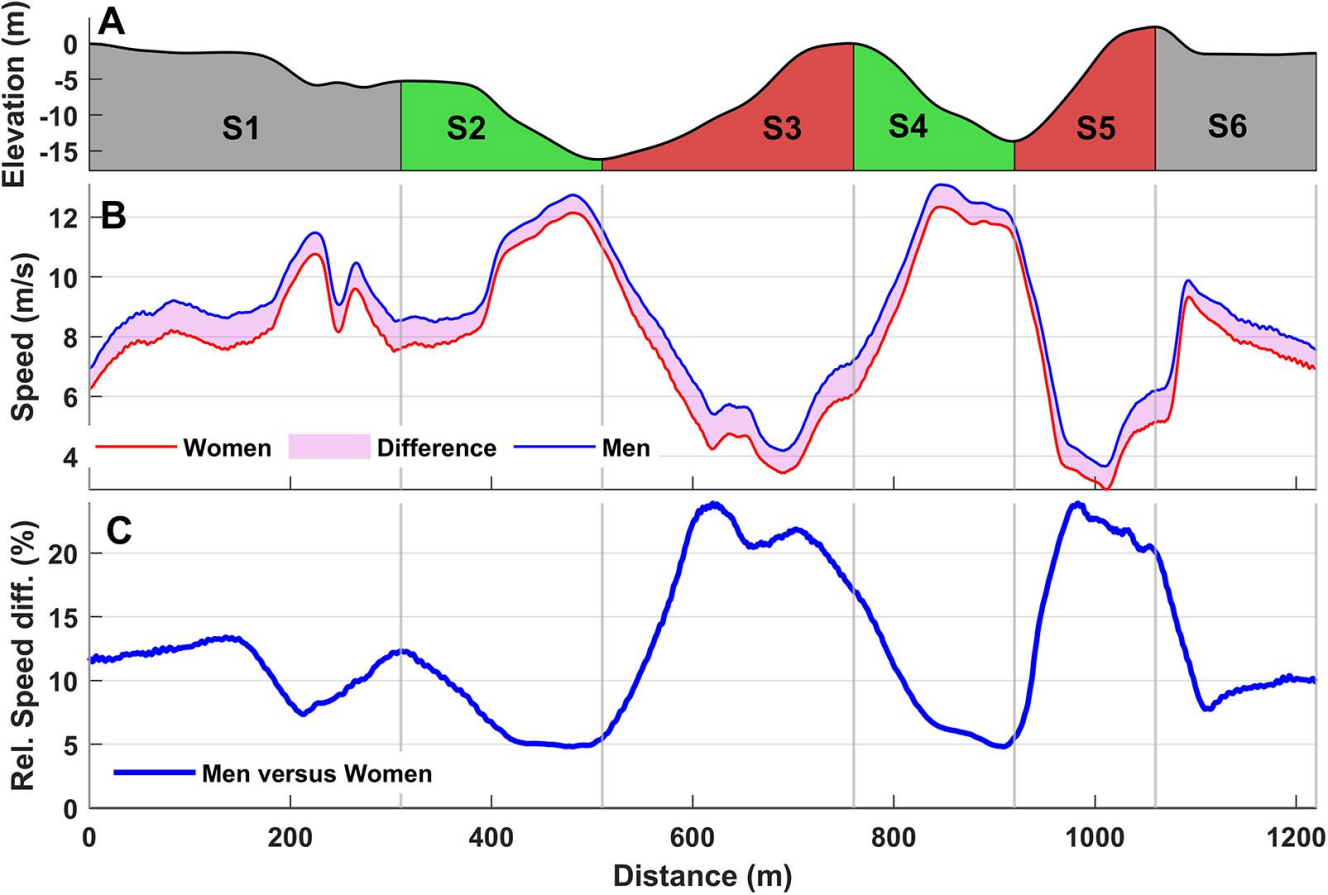
Elevation profile **(A)**, speed comparison **(B)**, and relative speed differences **(C)** between male and female skiers during a 1.3 km classical sprint cross-country skiing time-trial competition (1.3-km) divided into 6 different terrain sections (S1–6).

The competition was held under standardized conditions with temperatures ranging between −8 °C and −9 °C, with all participants starting within 38 min. Snow conditions were consistent throughout the competition, with a well-groomed racecourse composed of a mixture of natural and artificial snow. Wind speeds were less than 2 m/s, ensuring minimal external impact on skier performance. The racecourse (Figure 1) consisted of a balanced combination of uphill, flat, and downhill sections, with homologation data confirming adherence to FIS gradient standards.

### 2.4 Data collection and analysis

Time, speed, sub-technique selection, and cycle characteristics were continuously measured using an integrated GNSS and IMU unit (Optimeye S5, Catapult Innovations, Melbourne, Australia), which was secured in a customized bib on the participant's torso. The methodology used for data collection and processing followed previous protocols (8, 17). Each participant's GNSS track was aligned with a defined reference course to ensure consistency in data analysis.

### 2.5 Terrain and technique classification

The racecourse was segmented into predefined terrain segments (uphill, flat, and downhill) based on gradient thresholds defined in the FIS homologation manual (1). Uphill segments were defined as those with a climb of more than 10 meters and a gradient of more than 6%, while downhill segments were defined as those with a descent of more than 10 meters and a negative gradient of more than 6%. The remaining segments were classified as flat/undulating terrain segments, denoted as flat in the following. Sub-technique classification was performed using a Bidirectional Long Short-Term Memory (BiLSTM) neural network applied to the GNSS/IMU signals to automatically identify each skiing sub-technique. The classification model achieved an accuracy of 92%–94% for distinguishing sub-techniques on test datasets from an independent group of skiers who were not included in the data employed to develop the algorithms. Sub-techniques were classified according to Pellegrini et al. (16) as diagonal technique (DIA; in which our data included only a small amount of the herringbone technique), double poling with kick (DPK), double poling (DP), as well as combined tuck position and various turning techniques (TCK/TRN). Cycle characteristics were automatically segmented after classification based on peak detection of Gaussian low pass filtered data from the acceleration and gyroscope signals. Individual cycles were identified from recurrent IMU signal patterns, enabling calculation of cycle time (CT), cycle length (CL; distance traveled per cycle from GNSS data) and cycle rate (CR; cycles per minute from the inverse of cycle time) for each sub-technique. These parameters were analyzed separately for different terrain sections to investigate sex-based differences in efficiency and technique selection. Finally, due to the very small number of cycles using DPK, this data was omitted in the analysis.

### 2.6 Statistical analysis

All data were checked for normality using the Shapiro–Wilk test and visual inspection of QQ plots. The coefficient of variation (CV) for speed was calculated as the standard deviation (SD) divided by the mean and expressed as a percentage. An independent sample t-test was used to test for differences in FIS points and rank between male and female skiers. All data were analyzed using mixed general linear models (Python package statsmodels 0.14) with a random intercept for each participant identification. Different models were developed testing main effects of sex and terrain/segment/technique. Where appropriate, interaction terms (sex × factor) were included to test whether sex differences varied across conditions. Models were fitted using the Powell optimization method with restricted maximum likelihood (REML) estimation. Correlation analyses between total performance and performance in different terrain sections were performed using Pearson's product-moment correlation. Cohen d effect sizes were also calculated and interpreted according to Hopkins (18): 0−0.2 = trivial, 0.2−0.6 = small, 0.6−1.2 = moderate, 1.2−2.0 = large, and > 2 = very large. All statistical analyses were conducted in Python 3.13 using the libraries scipy (v1.x), statsmodels (v0.14.x), pandas (v2.x), and numpy (v1.x) with the level of statistical significance set at an alpha of < .05.

## 3 Results

### 3.1 Speed fluctuations and time distribution

Men demonstrated 14% higher average speeds compared to women (8.26 vs. 7.23 m/s, *P* < 0.001; see details in Supplementary Table S1). Speed differences between sexes varied across the six course segments, ranging from 6.8% to 20.3%. The largest differences occurred in the uphill segments (S3 and S5) while the smallest difference was observed in the first downhill segment (S2). Mixed linear model analysis confirmed significant main effects of both sex and segment on speed (both *P* < 0.001), with segment-specific terrain features (uphill vs. downhill) being the primary determinant of speed variation.

### 3.2 Speed and time distribution in different terrain types

The largest speed differences of 20% were evident in uphill terrain, compared to 12% and 8% for flat and downhill terrain, respectively (all *P* < 0.001; Table 2), with the effect sizes decreasing systematically from uphill to downhill terrain (Cohen's d: uphill = 5.39, flat = 4.73, downhill = 4.06). The continuous speed difference profile (Figure 1) demonstrates that sex differences in performance are inversely related to absolute speed, with the largest differences of up to 24% occurring in the steepest uphill sections where speeds were lowest, systematically decreasing to 10%–13% in flat terrain and 5%–8% in downhill sections where speeds were highest. Mixed linear model analysis revealed significant main effects of both sex (*P* < 0.001) and terrain (*P* < 0.001), with no significant sex-by-terrain interaction (*P* = 0.11), indicating that the sex difference in speed was relatively consistent across all terrain types. Correlations between speed in uphill, flat, and downhill terrain vs. total speed for women were *r* = 0.99, *r* = 0.92, and *r* = 0.88 (all *P* < 0.001), respectively, with corresponding values for men being *r* = 0.99, *r* = 0.89, and *r* = 0.91 (all *P* < 0.001). On average, women spent 2%-points more of their total time in uphill terrain (43.9% vs. 41.9%) and correspondingly spent less time in flat and downhill terrain compared to men (all *P* < 0.001; Table 2). Men spent 1.2%-points more time in downhill terrain (21.7% vs. 20.5%) compared to women.

**Table 2:** Speed, time and coefficient of variation for speed across terrain types during a classical sprint time-trial competition in elite- to world-class male and female cross-country skiers.

Terrain type	Variable	Women	Men	*P*-value	ES
Total	Time (s)	179.8 ± 3.5	157.5 ± 3.1	< 0.001	6.68
	Speed (m/s)	7.2 ± 0.1	8.3 ± 0.2	< 0.001	6.63
	CV (%)	2.0%	2.0%	Na	Na
Uphill	Time (s)	79.1 ± 2.0	66.1 ± 2.1	< 0.001	6.36
	Relative time (%)	43.9 ± 0.3	41.9 ± 0.6	< 0.001	Na
	Speed (m/s)	4.9 ± 0.1	5.9 ± 0.2	< 0.001	6.14
	CV (%)	2.5%	3.1%	Na	Na
Flat	Time (s)	64.0 ± 1.2	57.4 ± 1.0	< 0.001	5.91
	Relative time (%)	35.6 ± 0.2	36.4 ± 0.4	< 0.001	Na
	Speed (m/s)	7.3 ± 0.1	8.2 ± 0.1	< 0.001	5.92
	CV (%)	1.9%	1.8%	Na	Na
Downhill	Time (s)	36.9 ± 0.5	34.3 ± 0.6	< 0.001	4.83
	Relative time (%)	20.5 ± 0.3	21.7 ± 0.4	< 0.001	Na
	Speed (m/s)	9.7 ± 0.1	10.5 ± 0.2	< 0.001	4.78
	CV (%)	1.5%	1.6%	Na	Na

### 3.3 Sub-technique selection

On average, men spent 7.6%-points more of their total time in DP (60.9% vs. 53.3%) and correspondingly 6.7%-points less time in DIA (15.9% vs. 22.6%) compared to women (all *P* < 0.001; Table 3). Mixed linear model analysis revealed a significant sex-by-technique interaction (*P* < 0.001), indicating that the difference in technique selection between sexes varied depending on the specific technique investigated. These differences in technique selection were terrain-dependent (Table 4). In uphill terrain, women spent 54.5% of their time in DIA compared to 41.6% for men (*P* = 0.007), while men used DP for 54.7% vs. women's 39.7% (*P* = 0.003). On flat terrain, both sexes predominantly used DP (∼78%–80% of the time), with minimal use of DIA.

**Table 3:** Percentage of time spent in different sub-techniques and related cycle characteristics during a classical sprint time-trial qualification in elite- to world-class male and female cross-country skiers.

Category	Variable	Women	Men	*P*-value	ES
Time (%)	DIA	22.6 ± 3.8	15.9 ± 1.3	< 0.001	−2.35
Time (%)	DP	53.3 ± 5.8	60.9 ± 2.9	< 0.001	1.67
Time (%)	TCK/TRN	21.4 ± 3.0	20.7 ± 2.6	0.454	−0.28
CL (m)	DIA	2.89 ± 0.23	3.14 ± 0.35	0.027	0.86
CR (min^−1^)	DIA	77.4 ± 4.6	81.6 ± 6.1	0.041	0.78
CL (m)	DP	5.87 ± 0.36	6.54 ± 0.34	< 0.001	1.92
CR (min^−1^)	DP	72.7 ± 3.7	72.7 ± 2.4	0.955	0.02

**Table 4:** Percentage of time spent in different sub-techniques across different terrains during a classical sprint time-trial qualification in elite- to world-class male and female cross-country skiers.

Terrain	Technique	Women (% time)	Men (% time)	Difference	*P*-value	ES
Uphill	DIA	54.5 ± 17.7	41.6 ± 18.0	−12.9	0.007	−0.72
Uphill	DP	39.7 ± 18.6	54.7 ± 18.7	14.9	0.003	0.80
Uphill	TCK/TRN	5.2 ± 2.4	3.4 ± 2.8	−1.8	0.010	−0.69
Flat	DIA	0.3 ± 1.4	0.0 ± 0.0	−0.3	0.225	−0.32
Flat	DP	77.5 ± 7.8	79.9 ± 5.0	2.4	0.170	0.36
Flat	TCK/TRN	21.9 ± 7.3	19.9 ± 5.0	−2.0	0.223	−0.32
Downhill	DIA	0.0 ± 0.0	0.1 ± 0.7	0.1	0.326	0.26
Downhill	DP	40.4 ± 14.1	42.2 ± 11.1	1.9	0.567	0.15
Downhill	TCK/TRN	59.6 ± 14.1	57.6 ± 11.3	−2.0	0.545	−0.16

**Figure 2:**
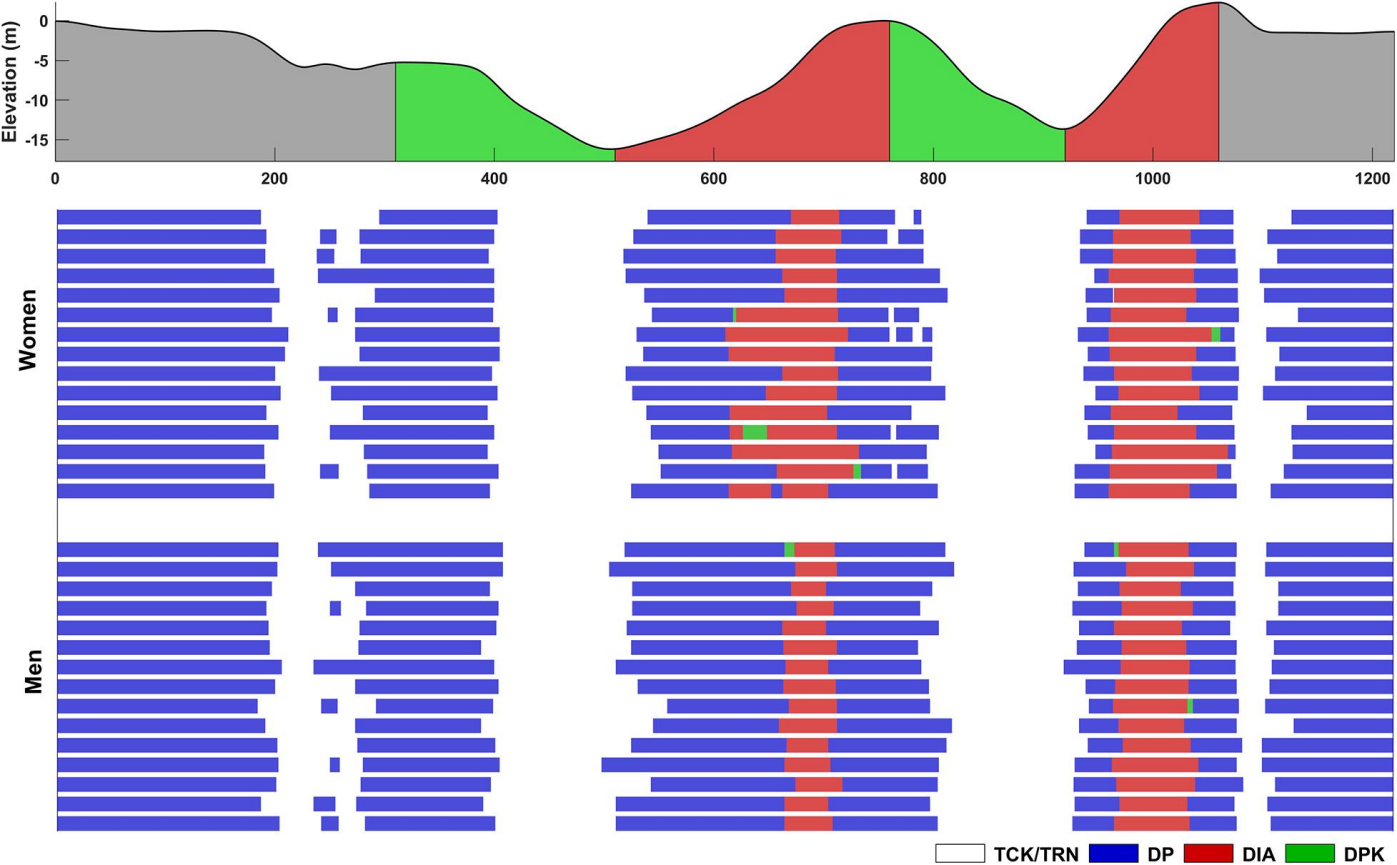
Individual sub-technique utilization for male and female skiers during a classical sprint cross-country skiing time-trial competition. Both males and females are ranked after performance, from top to bottom.

A closer examination of the uphill terrain revealed performance-related differences in technique selection. Among men, the fastest performers (*n* = 7) utilized DP for 61.2 ± 3.0% of their uphill time, compared to 56.5 ± 4.3% for the slower performers (*P* = 0.036, ES = 1.28). A similar trend was observed among women, with the fastest athletes using DP for 46.0 ± 9.1% of uphill time vs. 41.2 ± 10.1% for slower performers (*P* = 0.375, ES = 0.49), although this difference was not statistically significant. Individual analysis (Figure 2) showed that this pattern was especially pronounced in the longest uphill segment (S3) for women, where visual inspection indicated that the five fastest women exhibited DP usage patterns similar to those of the men; in total, seven out of fifteen women displayed this pattern.

**Figure 3:**
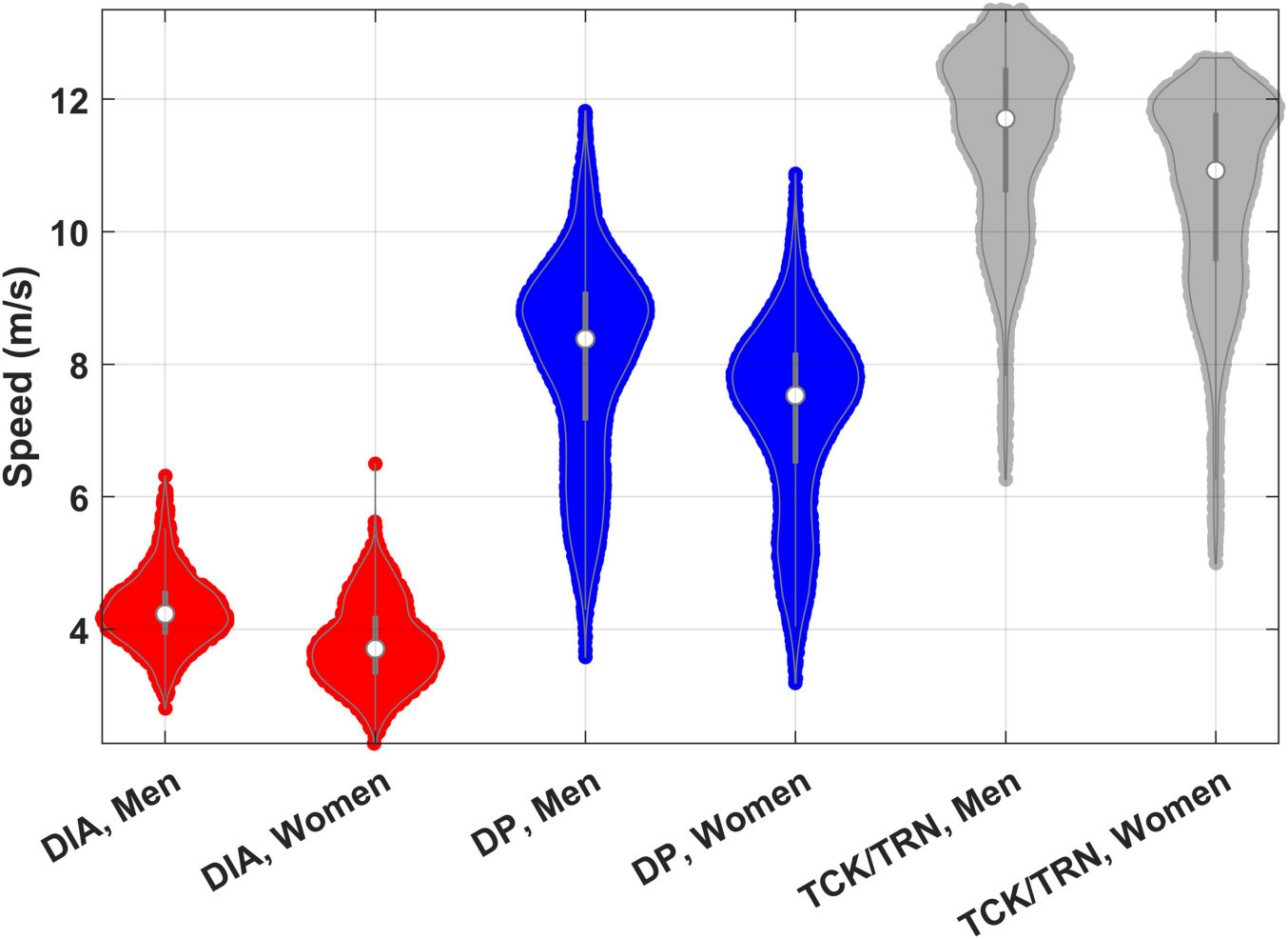
Speed distribution across sub-techniques for male and female skiers during a classical sprint cross-country skiing time-trial competition.

**Figure 4:**
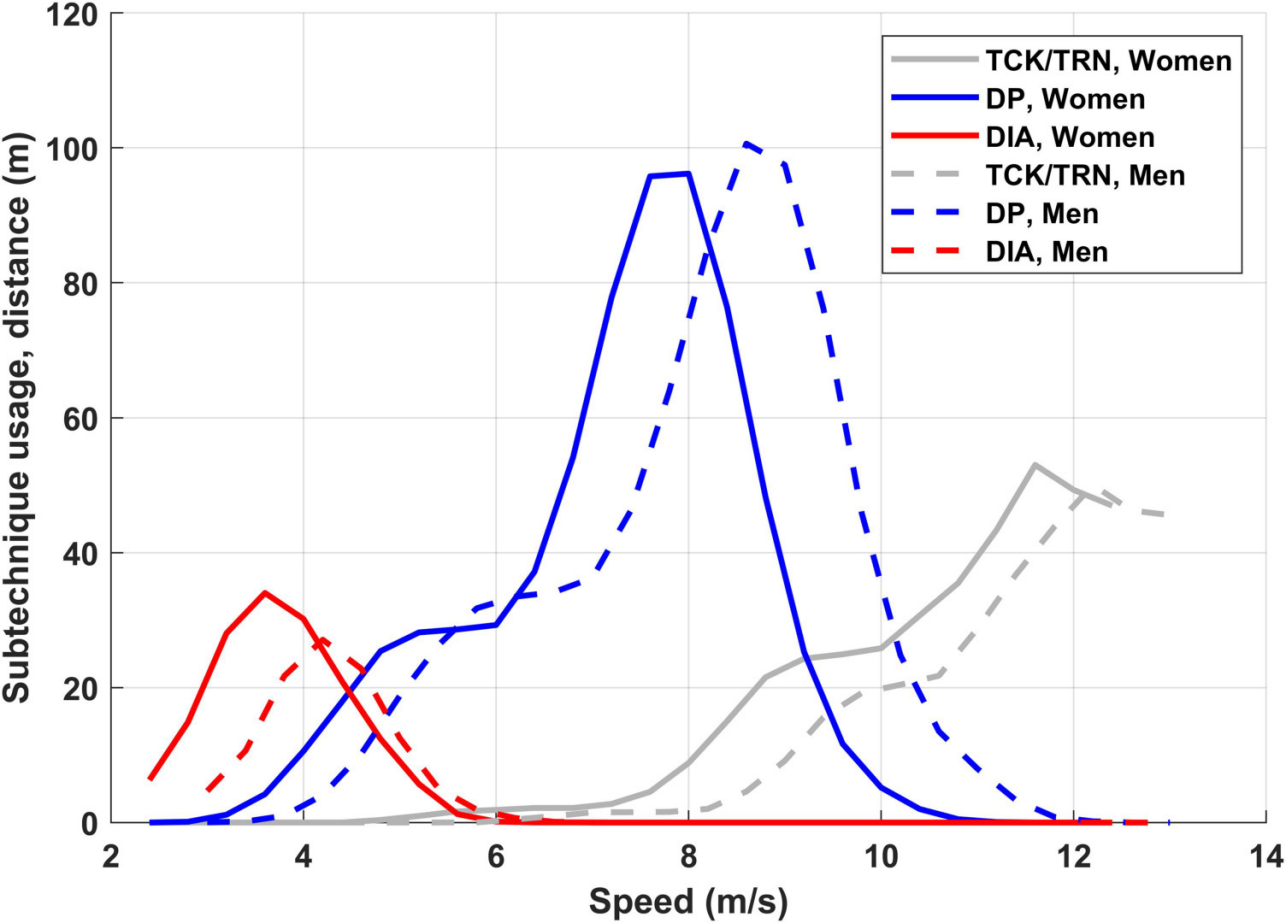
Sub-technique utilization as a function of speed for male and female skiers during a classical sprint cross-country skiing time-trial competition.

The speed distributions for each sub-technique (Figure 3) showed clear separation between sexes, with men executing all techniques at higher speeds. Figure 4 illustrates that technique transition speeds differed between sexes: women transitioned from DIA to DP at approximately 4–5 m/s, while men could maintain DP at lower speeds, extending further into uphill terrain. A corresponding sex difference was observed in the transition speed from DP to TCK/TRN.

### 3.4 Analysis of cycle characteristics

There were no significant differences between men and women in CR within the different sub-techniques (DP: 0.1% difference; DIA: 5.5% difference). However, men demonstrated approximately 8.8% and 11.4% longer CL in DIA and DP compared to women, respectively (both *P* < 0.001; Table 3). DP usage showed a moderate positive correlation with overall speed (*r* = 0.687, *P* < 0.001), while DIA usage showed a strong negative correlation with performance (*r* = −0.825, *P* < 0.001). Given that both sexes predominantly use DP on flat terrain, these correlations likely reflect technique selection differences in uphill sections, where the ability to maintain DP (rather than switching to DIA) appears to be a key performance differentiator. Terrain-specific analysis (Table 5) revealed that men's longer CL was consistent across all terrain types. In uphill DP, men achieved 14.1% longer cycles (5.47 vs. 4.79 m, *P* < 0.001), while in uphill DIA the difference was 10.5% (3.21 vs. 2.90 m, *P* = 0.010). Similar patterns were observed in flat terrain (12.4% difference) and downhill terrain (15.3% difference) for DP, with CR remaining largely similar between sexes across all conditions (no significant sex × terrain interactions in CR).

**Table 5:** Cycle characteristics for different sub-techniques across different terrains during a classical sprint time-trial qualification in elite- to world-class male and female cross-country skiers.

Terrain	Technique	Variable	Women	Men	Diff (%)	*P*-value	ES
Uphill	DIA	CL (m)	2.90 ± 0.36	3.21 ± 0.50	10.5%	0.010	0.69
Uphill	DIA	CR (min^−1^)	77.2 ± 5.9	80.9 ± 8.6	4.8%	0.061	0.50
Uphill	DP	CL (m)	4.79 ± 0.51	5.47 ± 0.36	14.1%	< 0.001	1.55
Uphill	DP	CR (min^−1^)	74.7 ± 3.6	74.6 ± 2.7	−0.1%	0.963	−0.01
Flat	DP	CL (m)	6.21 ± 0.44	6.98 ± 0.47	12.4%	< 0.001	1.69
Flat	DP	CR (min^−1^)	73.1 ± 4.7	73.1 ± 3.7	−0.0%	0.976	−0.01
Downhill	DP	CL (m)	6.78 ± 0.61	7.82 ± 0.55	15.3%	< 0.001	1.78
Downhill	DP	CR (cyc/min)	67.0 ± 5.0	65.8 ± 4.3	−1.7%	0.332	−0.25

## 4 Discussion

In this study, we found that male skiers were ∼14% faster than performance-matched female skiers during a classical sprint time-trial qualification performed across equal distance for both sexes. This difference was significant across all segments of the racecourse, with the largest disparity observed in uphill and the smallest in downhill segments. In addition, the men devoted a substantially greater portion of the competition to the DP sub-technique, whereas the women relied more on the DIA sub-technique. The men also achieved longer CL in both these sub-techniques, while CR remained comparable between sexes.

The observed speed differences between sexes in the sprint time-trial qualification are comparable to, or slightly higher than, those reported in other endurance sports (6, 7). Previous studies in cross-country skiing have reported that male skiers typically attain 10%–16% higher speeds than female skiers in distance competitions (8–11). It is notable that women spent more than 20 s more time overall, which in middle-distance events may influence the aerobic vs. anaerobic energy contribution significantly. Accordingly, men and women face different competitive demands when performing in similar courses. Here, the largest speed differences (and both absolute and relatively most time spent) between men and women occurred in uphill terrain, which aligns with previous literature demonstrating that the uphill segments are the most performance-differentiating in cross-country skiing (3, 9–11, 19). However, although the relative difference in speed was higher in uphill than downhill terrain, our analysis found no significant interaction between sex and terrain type. Since men entered downhill terrain with higher speed than women, which influenced the subsequent speed, the men maintained a performance advantage in all terrain types of the racecourse (uphill, flat, and downhill). Further, both sexes employed a similar pacing strategy, with a coefficient of variation in speed of only ∼2% throughout the race, indicating that men and women alike optimized their speed distribution in a comparable manner.

Another important finding is the clear sex difference observed in sub-technique selection during the time-trial qualification. The men spent approximately 61% of the total time using DP, compared to ∼53% for the women, and consequently devoted less time to DIA than the women. This disparity was especially pronounced in uphill terrain. On average, the women had to use DIA for over half of the time spent in uphill (∼55%), whereas the men limited DIA to around 42% of their uphill time (implying they could use DP for ∼58% of the uphill time). This observation is in line with previous findings that male skiers tend to select high-speed sub-techniques more often, while female skiers more frequently use slower techniques (10, 11). DP is generally the fastest classical sub-technique on flat to moderate uphill terrain, but it imposes greater demands on upper-body strength and endurance (3). It is therefore logical that men, who generally possess greater upper-body muscle mass and power, in addition to higher aerobic and anaerobic power, can sustain DP on inclines where women must transition to DIA to maintain forward propulsion (11, 12). Interestingly, the ability to maintain DP in uphill terrain also appears to be a discriminating factor within each sex. Faster men used significantly more DP than slower men in uphill terrain, with a similar trend observed among women. This suggests that speed is associated with the choice of sub-technique and that DP capacity in steep terrain represents a key difference between sexes as well as a performance characteristic that distinguishes faster from slower performers within both sexes. However, given the observational nature of this study, we cannot establish a causal relationship between sub-technique selection and speed; rather, both are likely influenced by underlying physiological differences (e.g., strength and power). A practical implication of the current findings is that female and male skiers have different sport-specific demands when competing at equal distances. Accordingly, training strategies should likely also reflect this difference in competitive demands.

The analysis of cycle characteristics provides further insight into how men achieve higher speeds compared to women, which further explains sex differences in sport-specific demands. Despite similar CR between sexes in both DP and DIA, the men attained significantly longer CL. In particular, the average distance covered per cycle in DP was about 0.7 m longer for men than for women (6.5 m vs. 5.8 m), and in DIA it was ∼0.2 m longer (3.1 m vs. 2.9 m). These differences correspond to roughly 10% longer distance per cycle for the men, which is in line with previous literature (8, 13). A longer CL indicates that the men produce greater propulsive impulse (force × time) per cycle, which is consistent with more controlled laboratory studies demonstrating that male elite skiers can generate higher poling forces than females (12–14), resulting in longer propulsion per cycle. Interestingly, CR did not differ notably between sexes (men's DIA CR was only ∼5% higher; *P* = 0.041, ES = 0.78), implying that both men and women adopted a similar CR for a given speed.

Men generally possess greater upper-body strength (20). For example, men exhibit a higher maximal shoulder torque capacity (21), which likely helps them maintain forceful DP on steep uphill sections where many women switch to DIA. In addition, men's larger body size provides greater muscle power and a possible mechanical advantage (greater ‘reach’), allowing them to apply force over longer distances, which may contribute to a longer CL compared to women. In contrast, the shorter limbs and lower upper-body strength of female skiers likely limit the peak impulse they can generate per stroke, particularly during steep uphill sections (12) This may lead to higher relative effort and faster fatigue in women, potentially explaining why they transition from DP to DIA sooner on steep terrain. Overall, these sex-based performance differences are not attributable to sheer ’strength’ alone but rather reflect a complex interaction between men's anthropometric advantages (longer levers) and greater upper-body power/torque capacity, which together enable a higher impulse per stroke.

Overall, this study provides new insight into sex differences in sprint cross-country skiing under the same racecourse conditions and with skiers of the same performance level. Men's higher speed leads to employment of faster sub-techniques and the ability to generate longer CL. These findings extend existing knowledge from distance competitions (8–11). Future research should also investigate how these sex differences develop in the subsequent heats of sprint competitions, and whether targeted training aimed at the identified factors can effectively improve performance among female sprinters.

### 4.1 Limitations and future directions

It should be noted that we did not measure individual anthropometrics (e.g., limb lengths) or upper-body strength in this study. Future studies should incorporate allometric scaling and direct measures of size and strength to better disentangle technique-related differences from those due to basic physical characteristics. Future research should also investigate how these sex-specific strategies influence fatigue over multiple sprint heats, e.g., whether men's high-impulse DP approach leads to greater fatigue or overuse strain than women's higher-frequency approach.

## 5 Practical applications

A practical implication of the current findings is that female and male skiers have different sport-specific demands when competing at equal distances during a sprint time-trial qualification. Female and male skiers utilize different biomechanical ‘tool kits’ to achieve high speeds, and training strategies should likely also be developed to reflect this difference. Rather than simply mimicking the male approach, female skiers may need a different solution for uphill speed; for instance, coaches could emphasize higher CR or employ targeted upper-body power training to improve DP performance.

Specifically, the most pronounced differences were found on uphill terrain, where men employed a greater proportion of DP and achieved longer CL in all main sub-techniques. Whether this implies that women should focus more on developing DIA or if they would benefit from focusing more on DP remains to be examined. The data indicate that both male and female skiers may benefit from technical training aimed at increasing CL (e.g., through more powerful and efficient push-offs) without compromising CR, as a longer CL at a given CR yields greater speed. However, technical efficiency should be evaluated relative to each skier's unique characteristics rather than against arbitrary standards. Lastly, the current findings also indicate that skiers capable of maintaining DP in steep uphill sections can gain a tactical advantage in sprint competitions.

## 6 Conclusions

Elite male skiers were ∼14% faster than performance-matched female skiers during a cross-country skiing sprint time-trial qualification. The most pronounced differences were found in uphill terrain, where men employed a greater proportion of DP and achieved longer CL in all the main sub-techniques. These results highlight DP capacity as a key performance differentiator in classical sprint cross-country skiing. While both sexes employed similar pacing strategies overall, the differences in upper-body strength and power likely a contributed to men achieving higher speeds and sustaining the DP sub-technique for longer.

## Data Availability

The datasets presented in this article are not readily available because individual athletes may be identified from the raw data. Requests to access the datasets should be directed to the corresponding author.
