# Supplementary material for: Sex differences in speed and sub-technique selection in elite sprint cross-country skiers: a time-trial qualification analysis
Source: Front Sports Act Living. 2026 Jun 17;8:1845432. doi: 10.3389/fspor.2026.1845432 (PMC13318749; doi:10.3389/fspor.2026.1845432)
Supplement: Supplementary file 1 [file Table1.docx]

**Supplementary tables**

| **Supplementary Table S1.** Speed, time and coefficient of variation for speed during a classical sprint time-trial competition in elite- to world-class male and female cross-country skiers. | | | | | | | |
| --- | --- | --- | --- | --- | --- | --- | --- |
| **Segment** | **Terrain** | **Variable** | **Women** | **Men** | **Diff (%)** | **P-value** | **ES** |
| S1 | Flat (310 m) | Speed (m/s) | 7.4 ± 0.2 | 8.2 ± 0.1 | 10.8% | < 0.001 | 4.99 |
|  |  | Time (s) | 42.1 ± 1.0 | 38.0 ± 0.7 | 10.8% | < 0.001 | 4.90 |
|  |  | CV (%) | 2.4% | 1.8% | - | Na | Na |
| S2 | Downhill (200 m) | Speed (m/s) | 9.7 ± 0.2 | 10.4 ± 0.2 | 6.8% | < 0.001 | 3.37 |
|  |  | Time (s) | 20.6 ± 0.4 | 19.3 ± 0.3 | 6.8% | < 0.001 | 3.35 |
|  |  | CV (%) | 2.1% | 1.7% | - | Na | Na |
| S3 | Uphill (250 m) | Speed (m/s) | 5.3 ± 0.1 | 6.3 ± 0.2 | 19.1% | < 0.001 | 6.23 |
|  |  | Time (s) | 47.6 ± 1.3 | 40.0 ± 1.1 | 19.1% | < 0.001 | 6.27 |
|  |  | CV (%) | 2.8% | 2.8% | - | Na | Na |
| S4 | Downhill (160 m) | Speed (m/s) | 9.8 ± 0.2 | 10.7 ± 0.2 | 9.0% | < 0.001 | 4.59 |
|  |  | Time (s) | 16.4 ± 0.3 | 15.0 ± 0.3 | 9.0% | < 0.001 | 4.66 |
|  |  | CV (%) | 1.6% | 2.1% | - | Na | Na |
| S5 | Uphill (140 m) | Speed (m/s) | 4.4 ± 0.2 | 5.4 ± 0.2 | 20.3% | < 0.001 | 4.56 |
|  |  | Time (s) | 31.5 ± 1.1 | 26.2 ± 1.2 | 20.3% | < 0.001 | 4.67 |
|  |  | CV (%) | 3.5% | 4.3% | - | Na | Na |
| S6 | Flat (160 m) | Speed (m/s) | 7.3 ± 0.2 | 8.2 ± 0.2 | 12.7% | < 0.001 | 4.67 |
|  |  | Time (s) | 22.0 ± 0.6 | 19.5 ± 0.5 | 12.7% | < 0.001 | 4.60 |
|  |  | CV (%) | 2.6% | 2.6% | - | Na | Na |
